# Cellular oxido-reductive proteins of *Chlamydomonas reinhardtii *control the biosynthesis of silver nanoparticles

**DOI:** 10.1186/1477-3155-9-56

**Published:** 2011-12-07

**Authors:** Indu Barwal, Peeyush Ranjan, Suneel Kateriya, Subhash Chandra Yadav

**Affiliations:** 1 Nanobiology Lab, Biotechnology Division, Council of Scientific and Industrial Research- Institute of Himalayan Bioresource Technology (CSIR-IHBT), Palampur, H.P. 176061, India; 2 Department of Biochemistry, University of Delhi South Campus, New Delhi, 110021, India

## Abstract

**Background:**

Elucidation of molecular mechanism of silver nanoparticles (SNPs) biosynthesis is important to control its size, shape and monodispersity. The evaluation of molecular mechanism of biosynthesis of SNPs is of prime importance for the commercialization and methodology development for controlling the shape and size (uniform distribution) of SNPs. The unicellular algae *Chlamydomonas reinhardtii *was exploited as a model system to elucidate the role of cellular proteins in SNPs biosynthesis.

**Results:**

The *C. reinhardtii *cell free extract (*in vitro*) and *in vivo *cells mediated synthesis of silver nanoparticles reveals SNPs of size range 5 ± 1 to 15 ± 2 nm and 5 ± 1 to 35 ± 5 nm respectively. *In vivo *biosynthesized SNPs were localized in the peripheral cytoplasm and at one side of flagella root, the site of pathway of ATP transport and its synthesis related enzymes. This provides an evidence for the involvement of oxidoreductive proteins in biosynthesis and stabilization of SNPs. Alteration in size distribution and decrease of synthesis rate of SNPs in protein-depleted fractions confirmed the involvement of cellular proteins in SNPs biosynthesis. Spectroscopic and SDS-PAGE analysis indicate the association of various proteins on *C. reinhardtii *mediated *in vivo *and *in vitro *biosynthesized SNPs. We have identified various cellular proteins associated with biosynthesized (*in vivo *and *in vitro) *SNPs by using MALDI-MS-MS, like ATP synthase, superoxide dismutase, carbonic anhydrase, ferredoxin-NADP^+ ^reductase, histone etc. However, these proteins were not associated on the incubation of pre-synthesized silver nanoparticles *in vitro*.

**Conclusion:**

Present study provides the indication of involvement of molecular machinery and various cellular proteins in the biosynthesis of silver nanoparticles. In this report, the study is mainly focused towards understanding the role of diverse cellular protein in the synthesis and capping of silver nanoparticles using *C. reinhardtii *as a model system.

## Background

Silver nanoparticles (SNPs) have extensive applications in civil, therapeutic and industrial areas as catalyst, cryogenic superconductor, biosensor, microelectronic and bacteriostatic materials [1-3], etc. These SNPs have been synthesized by various physical, chemical and biological methods. Among the various known synthesis methods, biosynthesis of silver nanoparticles is preferred as it is environmentally safe, low cost and less toxic [4]. These biologically synthesized silver nanoparticles (SNPs) could have better applications in therapeutics, drug delivery, anticancer and bio-imaging techniques. It has been known for the long time that in nature a variety of nanomaterials were synthesized by biological machinery. For example, the magneto-tactic bacteria synthesize intracellular magnetite nanocrystallites [5], diatoms synthesize siliceous materials [6], S-layer bacteria produce gypsum/calcium carbonate layers [7] and plants (algae, fungi, gymnosperms and angiosperms) for gold and silver nanoparticles [8]. In the past few years, bio-production of size and shape controlled SNPs has become a new and interesting research focus of the field [9-11]. The size and shape control of biosynthesized SNPs could be achieved by appreciative workout of bio-molecular and biochemical mechanism of SNPs biosynthesis. However, the biosynthesis mechanism of these silver nanoparticles by bio-molecular reduction and stabilization are still elusive. Some reports have highlighted the mechanism of metallic nanoparticles biosynthesis from different biological extract *in vitro*[12-14]. The overall biosynthesis of silver nanoparticles is reported as two-step reaction [8]. The first step involves trapping of Ag^+ ^inside the cell cytoplasm. In the second step, enzymes present in the cells are responsible for the reduction of silver ions. However, these investigators had not delineated the molecular machinery and involvement of biomolecules for SNPs biosynthesis. In another report, the presence of hydrogenase in the *F. oxysporum *broth was assumed to be responsible for the biosynthesis of SNPs [15-17]. This extra cellular enzyme shows excellent redox properties and it can act as an electron shuttle in metal reduction [15,18]. It was evident that electron shuttles or other reducing agents (e.g., hydroquinones) released by microorganisms are capable of reducing ions to nanoparticles [19]. However, the possible involvements of various macromolecules for the reduction of silver ion into silver nanoparticles are not reported yet. The identification of macromolecules responsible for the SNPs biosynthesis would delineate the possible mechanism of SNPs production in biological model systems.

Here we report, for the first time, the involvement of various proteins of oxido-reductive system and nuclear histone protein of *C. reinhardtii *along with biochemical substantiation in the biosynthesis and capping of SNPs. The silver nanoparticles associated proteins were identified by mass spectrometry and the synthesized silver nanoparticles were characterized by transmission electron microscope (TEM) for their shape and size distribution. The current research report proposes the possible molecular basis of the biosynthesis of SNPs and the involvement of the oxidoreductive machinery in biosynthesis and stabilization of silver nanoparticles using *C. reinhardtii *as model system.

## Results

### Biosynthesis of silver nanoparticles (SNPs)

**Figure 1:**
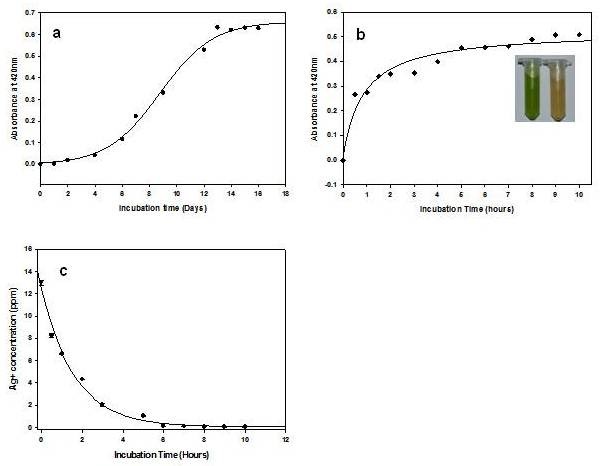
**Kinetics of biosynthesis of silver nanoparticles **(a) Synthesis of nanoparticles in *C. reinhardtii *cell free extracts (*in vitro*) system and (b) *in vivo *condition. The color of the *C. reinhardtii *cell becomes yellowish brown after incubation with 1 mM AgNO_3 _in 5 hours (inset). (c) ICP-MS quantification of remaining Ag^+ ^(in ppm) after removal of *in vivo *biosynthesized SNPs.

*In vitro *synthesis of SNPs by *C. reinhardtii *cell free extract was very slow (completed in 13 days) and synthesis kinetics was sigmoidal (Figure 1a). *In vivo *biosynthesis of SNPs was comparatively much faster than that of *in vitro *condition (Figure 1b). The biosynthesis was completed within 10 h of incubation *in vivo *(Figure 1b). The reduction of Ag^+ ^into Ag^0 ^(SNPs) has appeared just after the incubation and half of the SNPs were synthesized within 30 min. The color of the culture solution turned yellowish brown while the control algal culture (without AgNO_3_) remained green (Figure 1b inset). The synthesis of silver nanoparticles by *C. reinhardtii *live cell showed hyperbolic synthesis kinetics (Figure 1b). The maximum level of reduced silver (metal nanoparticles) in *C. reinhardtii *(*in vitro *and *in vivo*) was in the order of ~0.5-0.6 absorbance unit (Additional File 1). It was found that the amount of SNPs has an upper limit, which depends on the reducing capacity of the biological extracts, AgNO_3 _concentration and incubation time for the ease of reduction of the metal (Figure 1).

### Morphological characterization of biosynthesized SNPs

**Figure 2:**
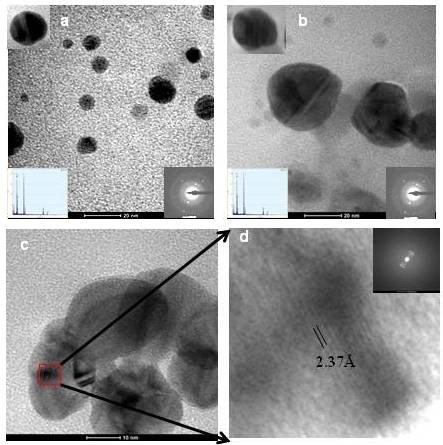
**Morphological characterization of the silver nanoparticles**. (a) *In vitro *synthesis, diffraction pattern, and EDAX analysis was given in inset (b) *In vivo *synthesis and its diffraction pattern (inset) (c) TEM high resolution Image (HR Image) of *C. reinhardtii *cell mediated synthesized silver nanoparticles and (d) Magnified view of SNPs fringes and Fourier transform image in inset.

Transmission electron microscopy (TEM) was used for morphological characterization of *in vitro *and *in vivo *synthesized SNPs. The shape of the biosynthesized SNPs in both conditions were rounded/rectangular. However, the size and universal size distribution were different for *in vitro *and *in vivo *biosynthesized SNPs. *In vivo *biosynthesized SNPs were relatively larger than *in vitro *biosynthesized silver nanoparticles. The *in vitro *synthesized nanoparticles were of size range 5 ± 1 to 15 ± 2 nm (Figure 2a) whereas, the sizes range of *in vivo *biosynthesized nanoparticles were 5 ± 1 to 35 ± 5 nm (Figure 2b).The crystalline nature of these nanoparticles was further confirmed by using TEM selected area distribution study (SADS). The bright spot at regular interval confirms the highly crystalline nature of biosynthesized silver nanoparticles (Figure 2a &2b inset). The EDAX (Energy-dispersive X-ray spectroscopy) analysis also confirms the presence of silver atoms in biosynthesized nanoparticles (Figure 2a &2b inset). High-resolution (HR) and Fourier transform images are the confirmatory signature for crystalline nature of biosynthesized nanoparticles (Figure 2c and 2d).

### Localization of *in vivo *synthesized SNPs in *C. reinhardtii *cell

**Figure 3:**
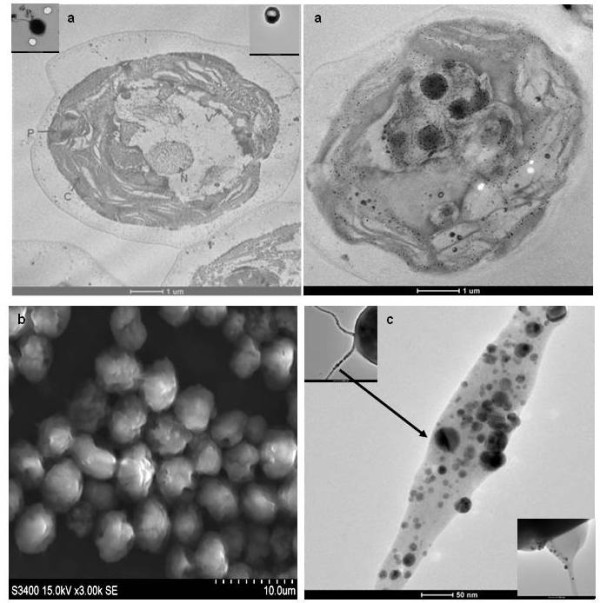
**Cellular localization of *in vivo *synthesized silver nanoparticles **(a) TEM micrograph of thin section (~60 nm) and (b) SEM image of 1 mM AgNO_3 _incubated *C. reinhardtii *cell. (c) Silver nanoparticles localized on the flagellum.

The black regions in the cytoplasm of *C. reinhardtii *cells were due to the biosynthesis of silver nanoparticles, which were gradually increased and finally filled the complete cell (opaque cell, Figure 3a inset). Similar patterns were also observed by scanning electron microscope (SEM) analysis (Figure 3b). Normal *C. reinhardtii *cells after incubation with AgNO_3 _show brighter spot inside the cell due to synthesis of silver nanoparticles (Additional File 2). The SNPs were densely localized in periplasm and cytoplasm in the thin section (~ 60 nm) of *in vivo *biosynthesized SNPs containing cells (Figure 3a and 3b and Additional File 3). On the closer evaluation of the nanoparticles distribution within flagella of *C. reinhardtii *cell, it was found that these SNPs were also distributed inside and outside the flagella. The numbers of nanoparticles were more towards the basal body end than the distal end of the flagella (Figure 3c and inset). The SNPs were highly localized at one side of flagella root (basal body) and some part of the flagella was distended due to the presence of excessive amount of nanoparticles internally (Figure 3c and inset).

### Protein depletion and its effect on rate of nanoparticles synthesis, morphology and size

**Figure 4:**
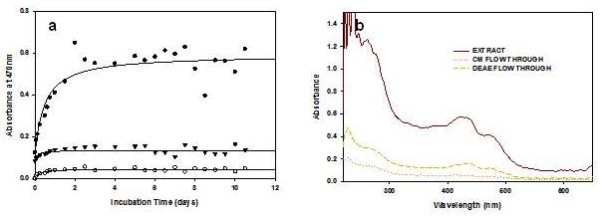
**Total protein depletion experiments of *C. reinhardtii *cell free extract **(a) Synthesis kinetics of SNPs by *C. reinhardtii *cell free extract (●), DEAE-sepharose (▼), CM-sepharose (○) depleted flow through samples (b) The absorbance scans of *C. reinhardtii *cell free extract, DEAE-sepharose and CM-sepharose depleted flow through samples after silver nanoparticles synthesis.

Proteins depletion experiments were performed to confirm the role of cellular proteins in the control of synthesis rate of SNPs and their morphology. Cell free extract was depleted on pre-equilibrated anion (DEAE-sepharose) and cation exchange (CM-sepharose) chromatography columns at pH 7.0 to maintain the functional and physiological condition of cellular proteins. The SDS-PAGE and absorption scan of extract, DEAE and CM-sepharose depleted fraction confirmed the various degree of protein depletion (Additional File 4 and 5). The *C. reinhardtii *cell free extract showed multiple bands, few bands in DEAE-sepharose depleted solution, and no detectable band in CM-sepharose eluted fractions (Additional File 4). The absorption peaks of these eluted fractions were recorded at 280 nm and their intensity were higher for extract, lower for DEAE-sepharose and lowest for CM-sepharose eluted fractions (Additional File 5). The flow through of each column and cell free extract was incubated with Ag^+ ^and the synthesis kinetics was measured by absorption spectroscopy. The *C. reinhardtii *cell free extract showed fast synthesis in comparison to that of ion exchange chromatography depleted fractions (Figure 4a and 4b). CM-sepharose eluted fraction showed lowest synthesis among all the fractions (Figure 4a and 4b).

**Figure 5:**
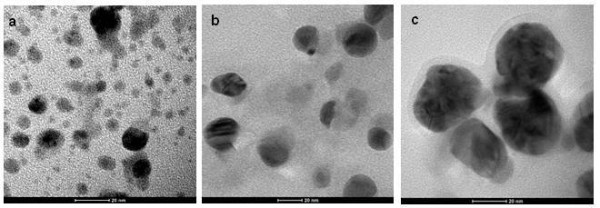
**Morphological characterization of protein depleted synthesized SNPs **(a) *C. reinhardtii *cell free extract, (b) DEAE-sepharose and (c) CM-sepharose depleted flow through solution synthesized SNPs.

The effect of protein depletion on size and morphology was also evaluated by the transmission electron microscope (TEM). The size of nanoparticles synthesized by extract was similar (5.0 ± 0.5 to ≥ 15 ± 2 nm) as reported (Figure 2a and 5a). However, the size of nanoparticles synthesized by DEAE-sepharose flow through (20 ± 4 nm) was larger in comparison to *C. reinhardtii *cell free extract (Figure 5b). CM-sepharose flow through fraction resulted 27 ± 5 nm silver nanoparticles synthesis under the similar conditions (Figure 5c).

### Identification of SNPs bound proteins

**Figure 6:**
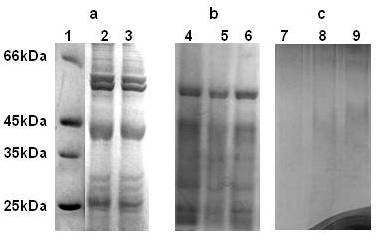
**Cellular protein profiling of biosynthesized SNPs**. Biosynthesized silver nanoparticles were gently washed (DW) and equal amount of bound proteins were loaded on reducing SDS-PAGE. The freshly synthesized silver nanoparticles (protein and surfactant free) were incubated with *C. reinhardtii *cell free extracts for three days. (a) Molecular weight marker (lane no 1), crude extract (lane no 2) and supernatant after nanoparticles synthesis (lane no 3). (b) Silver nanoparticles bound protein after 4 days (lane no 4), 7 days (lane no 5) and 12 days (lane no 6) incubation at reaction condition. (c) Protein binding on pre-synthesized silver nanoparticles of increasing amount 40 μl (lane7), 70 μl (lane 8) and 100 μl (lane 9). The washed isolated nanoparticles were subjected for SDS-PAGE analysis with different increasing volume.

Proteins associated on the *in vitro *and *in vivo *synthesized SNPs were resolved on SDS-PAGE and the protein bands were processed for MALDI-MS and MALDI-MS-MS analysis (Figure 6a and 6b). The SNPs associated proteins of *in vitro, in vivo*, DEAE and CM-sepharose depleted fraction on SDS-PAGE were separately analyzed by MALDI MS-MS *de novo *sequencing. Nearly 18 silver nanoparticles bound proteins were identified from various biosynthesis conditions. Most of the identified SNPs associated proteins (*in vitro *and *in vivo *condition) were the part of oxido-reductive machinery and showed involvement in ATP synthesis, photosystem, and stress response (Additional File 6). The MS-MS and mascot search details of the SNPs associated bound proteins were summarized in the Table 1. The freshly synthesized and thoroughly washed (to remove proteins and other bound surfactants) silver nanoparticles were incubated with *C. reinhardtii *cell free extracts for 3.0 days at room temperature. Separated and washed pre-synthesized SNPs reveal different protein bands patterns to that of biosynthesized SNPs on SDS-PAGE (Figure 6c).

**Table 1:** MALDI-TOF-TOF of various SNPs associated proteins with matched sequences

**No**.	Protein Name	MW	Coverage (%)	Peptide	Full proteins Sequence (Matched peptides bold underlined)
1	Histone H4	11.4	19.4	R.ISGLIYEETR.T; K.TFLENVIR.D; R.DNIQGITKPAIR.R; K.RISGLIYEETR.T	MSGRGKGGKGLGKGGAKRHRKVL**RDNIQGITKPAIRR**LARRGGV**KRISGLIYEETRT**VL**KTFLENVIR**DSVTYTEHARRKTVTAMDVVYALKRQGRTLYGFGG

2	Carbonic anhydrase	41.6	42.8	K.QSPINVPQYQVLDGK.G; R.IVDVLEMRPNDAADR.V R.VTAVPTQFHFHSTSEHLLAGK.I; K.IYPLELHIVHQVTEK.L; R.EGTFSNLPAGTTIK.L K.LGELLPSDR.D; R.ISFGQWNR.Y K.ATVSGDHWDHGLNGENWEGK.D R.DYVTYEGSLTTPPCSEGLLWHVMTQPQR.I	CIYKFGTSPDS**KATVSGDHWDHGLNGENWEGKD**GAGNAWVCKTGRKQSPINVPQYQVLDGKGSKIANGLQTQWSYPDLMSNGTSVQVINNGHTIQVQWTYNYAGHATIAIPAMHNQTN**RIVDVLEMRPNDAADRVTAVPTQFHFHSTSEHLLAGKIYPLELHIVHQVTEKL**EACKGGCFSVTGILFQLDNGPDNELLEPIFANMPS**REGTFSNLPAGTTIKLGELLPSDRDYVTYEGSLTTPPCSEGLLWHVMTQPQRISFGQWNRY**RLAVGLKECNSTETAADAGHHHHHRRLLHNHAHLEEVPAATSEPKHYFRRVMLAESANPDAYTCKAVAFGQNFRNPQYANGRTIKLARYH

3	Ferredoxin--NADP reductase	39.2	8.4	K.VLLLPADANAPLICVATGTGIAPFR.S	AATKASTAVTTDMSKRTVPTKLEEGEMPLNTYSNKAPFKAKVRSVEKITGPKATGETCHIIIETEGKIPFWEGQSYGVIPPGTKINSKGKEVPTARLYSIASSRYGDDGDGQTASLCVRRAVYVDPETGKEDPAKKGLCSNFLCDATPGTEISMTGPTG**KVLLLPADANAPLICVATGTGIAPFR**SFWRRCFIENVPSYKFTGLFWLFMGVGNSDAKLYDEELQAIAKAYPGQFRLDYALSREQNNRKGGKMYIQDKVEEYADEIFDLLDNGAHMYFCGLKGMMPGIQDMLERVAKEKGLNYEEWVEGLKHKNQWHVEVY

4	Superoxide dismutase	23.9	10.1	R.RPEYIAAWWNVVNWEQVAENYK.A	MAQALPPLPYDYGSLEPHVDATTMNIHHTKHHQTYVNNLNAALDKFPELKDLGLVDLNKAVGTDKLPKDVATVIRNNGGGHYNHSFFWKVMTNPSNTNGPNGDVKAAIEASFGSVDEMKAKFNAAAAGRFGSGWAWLSVKPDGSLSIDSTPNQDNPLMTALPDVAGGIPLLGLDVWEHAYYLKYQNR**RPEYIAAWWNVVNWEQVAENYKA**AQAGTVPL

5	Sedoheptulose-1,7-bisphosphatase	41.7	21.8	R.ATFDNPAYER.L K.AVSALDIPILVCDQR.T R.TQICYGSIGEVR.R R.EQVAAGMGIYGPR.T R.LINFYLGEK.Y R.ILFEVAPLALLIEK.A	RAARVQSRRTAVLTQAKIGDSLAEFLVEATPDPKLRHVMMSMAEATRTIAHKVRTASCAGTACVNSFGDEQLAVDMVADKLLFEALKYSHVCKLACSEEVPEPVDMGGEGFCVAFDPLDGSSSSDTNFAVGTIFGVWPGDKLTNITG**REQVAAGMGIYGPRT**VFCIALKDAPGCHEFLLMDDGKWMHVKETTHIGEGKMFAPGNL**RATFDNPAYERLINFYLGEKY**TLRYTGGIVPDLFQIIVKEKGVFTNLTSPTTKAKL**RILFEVAPLALLIEKA**GGASSCDG**KAVSALDIPILVCDQRTQICYGSIGEVR**RFEEYMYGTSPRFSEKVVA

6	ATP synthase subunit alpha	54.7	53.5	K.MVDFGIVFQVGDGIAR.I; K.IAEIPVGEAYLGR.V R.VVDGLARPVDGK.G; R.AIESPAPGIVAR.R R.SVYEPLATGLVAVDAMIPVGR.G K.TAIAVDTILNQK.G; K.ASSVAQVLNTLKER.G R.EAYPGDVFYLHSR.L K.LKLELAQFAELEAFSQFASDLDQATQNQLAR.G R.STLTFTPEAEGLVK.Q; K.QAINEYLEEFKSQAK.A R.TPEELSNLIKDLIEQYTPEVK.M; K.QAQAYREMSLLLR.R; K.DLIEQYTPEVK.M; K.GVICVYVAIGQK.A; K.QPQSSPLSVEEQVASLYAGTNGYLDKLEVSQVR.A	MAM**RTPEELSNLIKDLIEQYTPEVKMVDFGIVFQVGDGIARI**YGLEKAMSGELLEFEDGTLGIALNLEANNVGAVLLGDGLKITEGSRVRCTG**KIAEIPVGEAYLGRVVDGLARPVDGKG**AVQTKDS**RAIESPAPGIVARRSVYEPLATGLVAVDAMIPVGRG**QRELIIGDRQTG**KTAIAVDTILNQKGKGVICVYVAIGQKASSVAQVLNTLKERG**ALDYTIIVMANANEPATLQYLAPYTGATLAEYFMYTGRPTLTIYDDLS**KQAQAYREMSLLLR**RPPG**REAYPGDVFYLHSRL**LERAAKLNNALGEGSMTALPIVETQEGDVSAYIPTNVISITDGQIFLAAGLFNSGLRPAINVGISVSRVGSAAQPKAMKQVAG**KLKLELAQFAELEAFSQFASDLDQATQNQLARG**ARLREIL**KQPQSSPLSVEEQVASLYAGTNGYLDKLEVSQVRA**YLSGLRSYLANSYPKYGEIL**RSTLTFTPEAEGLVKQAINEYLEEFKSQAKA**A

7	ATP synthase subunit beta	52.0	30.9	K.GQVPNIYNALTIR.A; R.TAPAFVDLDTRLSIFETGIK.V R.LSIFETGIK.V; K.VVDLLAPYR.R K.VVDLLAPYRR.G; K.IGLFGGAGVGK.T K.AHGGVSVFAGVGER.T; K.VALVYGQMNEPPGAR.M R.DVNKQDVLFFIDNIFR.F; R.FVQAGAEVSALLGR.M R.TAPAFVDLDTR.L; R.VALTALTMAEYFR.D R.DVNKQDVLFFIDNIFR.F; K.TVLIMELINNIAK.A K.QDVLFFIDNIFR.F	MSDSIETKNMGRIVQIIGPVLDIVFA**KGQVPNIYNALTIRA**KNSAGTEMAVTCEVQQLLGDNCVRAVSMNPTEGLMRGMEVVDTGKPLSVPVGKVTLGRIFNVLGEPVDNMGNVKVEETLPIH**RTAPAFVDLDTRLSIFETGIKVVDLLAPYRRGGKIGLFGGAGVGKTVLIMELINNIAKAHGGVSVFAGVGERT**REGNDLYTEMKESGVIVEKNLSDS**KVALVYGQMNEPPGARMRVALTALTMAEYFRDVNKQDVLFFIDNIFRFVQAGAEVSALLGRM**PSAVGYQPTLATEMGGLQERITSTKDGSITSIQAVYVPADDLTDPAPATTFAHLDATTVLSRNLAAKGIYPAVDPLESTSTMLQPWILGEKHYDSAQSVKKTLQRYKELQDIIAILGLDELSEEDRLIVARARKIERFLSQPFFVAEVFTGSPGKYVSLAETIEGFGKIFAGELDDLPEQAFYLVGNITEAISKAASLK

8	ATP synthase gamma chain	38.7	15.0	K.VLYGVNQR.V; R.SLQEALASELAAR.M R.AQEAVVNGRPFSENLVK.V K.SVLLVVLTGDR.	MAAMLASKQGAFMGRSSFAPAPKGVASRGSLQVVAGLKEVRDRIASVKNTQKITDAMKLVAAAKVR**RAQEAVVNGRPFSENLVKVLYGVNQRV**RQEDVDSPLCAVRPV**KSVLLVVLTGDR**GLCGGYNNFIIKKTEARYRELTAMGVKVNLVCVGRKGAQYFARRKQYNIVKSFSLGAAPSTKEAQGIADEIFASFIAQESDKVELVFTKFISLINSNPTIQTLLPMTPMGELCDVDGKCVDAADDEIFKLTTKGGEFAVEREKTTIETEALDPSLIFEQEPAQILDALLPLYMSSCLL**RSLQEALASELAARM**NAMNNASDNAKELKKGLTVQYNKQRQAKITQELAEIVGGAAATSG

9	Oxygen evolving enhancer protein (OEE)1	30.5	20.8	R.VAFLFTIK.Q K.VTGLWYAQLK K.VGSDGSAELKEDDGIDYAATTVQLPGGER.V	LTFDEIQGLTYLQVKGSGIANTCPVLESGTTNLKELKAGSYKLENFCIEPTSFTVKEESQFKGGETEFVKTKLMTRLTYTLDAMSGSF**KVGSDGSAELKEDDGIDYAATTVQLPGGERVAFLFTIKQ**FDGKGTLDGIKGDFLVPSYRGSSFLDPKGRGGSTGYDNAVALPARADAEELLKENVKITKALKGSAVFSVAKVDPVTGEIAGVFESIQPSDTDLGAKPPKDI**KVTGLWYAQLK**

10	OEE2	25.8	25.5	K.WNPSKENDFPGVILR.Y; K.ENDFPGVILR.Y K.QAYSGETQSEGGFAPNR.V; K.TYYKYELLVR.S	AYGDSANVFGKVTNKSGFVPYAGDGFALLLPA**KWNPSKENDFPGVILRY**EDNFDAVNNLVVIAQDTDKKAIADFGSQDKFLESVSYLLG**KQAYSGETQSEGGFAPNRV**SAASLLDVSTTTDKKG**KTYYKYELLVRS**ADGDEGGRHQLIGATVGSDNKLYIIKIQIGDKRWFKGAKKEAMGAFDSFTVV

11	OEE-3	21.8	25.6	K.EFIQAVEDLDFALR.E; R.DRGFDLIYEAR.D R.GFDLIYEAR.D; R.DLDLPQNVR.E	LTPVDLFDDRSV**RDRGFDLIYEARDLDLPQNVRE**GFTQARASLDETKKRVKESEARIDADLDVFIQKSYWTEAREQLRRQVGTLRFDLNTLASTKEKEAKKAALGLR**KEFIQAVEDLDFALRE**KDQASAAKKLEITKAKLDSVLAAVL

12	Ribulose bisphosphate carboxylase (RuBisCO) (LC)	52.5	2.5	R.FLFVAEAIYK.A	TKAGAGFKAGVKDYRLTYYTPDYVVRDTDILAAFRMTPQLGVPPEECGAAVAAESSTGTWTTVWTDGLTSLDRYKGRCYDIEPVPGEDNQYIAYVAYPIDLFEEGSVTNMFTSIVGNVFGFKALRALRLEDLRIPPAYVKTFVGPPHGIQVERDKLNKYGRGLLGCTIKPKLGLSAKNYGRAVYECLRGGLDFTKDDENVNSQPFMRWRD**RFLFVAEAIYKA**QAETGEVKGHYLNATAGTCEEMMKRAVCAKELGVPIIMHDYLTGGFTANTSLAIYCRDNGLLLHIHRAMHAVIDRQRNHGIHFRVLAKALRMSGGDHLHSGTVVGKLEGEREVTLGFVDLMRDDYVEKDRSRGIYFTQDWCSMPGVMPVASGGIHVWHMPALVEIFGDDACLQFGGGTLGHPWGNAPGAAANRVALEACTQARNEGRDLAREGGDVIRSACKWSPELAAACEVWKEIKFEFDTIDKL

13	RuBisCO (SC)	20.6	39.3	K.AYVSNESAIR.F; R.FGSVSCLYYDNR.Y K.AFPDAYVR.L; K.QVQIMGFLVQRPK.T R.DFQPANKR.S	MMVWTPVNNKMFETFSYLPPLTDEQIAAQVDYIVANGWIPCLEFAEAD**KAYVSNESAIRFGSVSCLYYDNR**YWTMWKLPMFGCRDPMQVLREIVACT**KAFPDAYVR**LVAFDNQ**KQVQIMGFLVQRPK**TA**RDFQPANKR**SV

## Discussion

An important area of research in nanotechnology deals with the controlled synthesis of environmentally benign nontoxic nanoparticles of desired shape, sizes and monodispersity. As a result, researchers in the field of nanoparticles synthesis have turned to biological systems to achieve desired qualities in synthesized nanoparticles. Many organisms (both unicellular and multi-cellular) are able to produce nanomaterials either intra- or extra-cellularly, which are mediated by various oxidoreductive molecules in the cells [16,20-27]. However, the molecular identity and role of these biomolecules in SNPs biosynthesis are yet to be reported. Our experiment shows that *C. reinhardtii *cells (*in vivo*) mediated biosynthesis of SNPs is faster than that of the *in vitro *(with cell free extract) condition (Figure 1a and 1b). This is due to the presence of different active biomolecules in the live cells (*in vivo*) than the cell free extract. *In vivo *synthesized nanoparticles are localized in cytoplasm, periplasm, nucleus, pyrenoids, and also in the flagella assembly as reported for the *S. algae *[7,28,29](Figure 3). Incubation of the pre-synthesized silver nanoparticles *in vitro *does not bind any protein while under similar condition the biosynthesized SNPs does (Figure 6b and 6c). This confirms that these proteins only bind to SNPs during biosynthesis and it does not bind on the pre-synthesized silver nanoparticles surface. These findings indicate that the active proteins in cells play major role in the silver nanoparticles biosynthesis.

Most of the *C. reinhardtii *cellular protein has been depleted with CM- and DEAE-sepharose ion exchange columns (Additional File 3). The proteins depleted fractions show large size SNPs and slower synthesis (Figure 4). This is an indication of direct involvement of active proteins in SNPs synthesis. It was observed that the size of biosynthesized SNPs is inversely proportional to the total protein content. This might be one of the reasons for the synthesis of large-sized silver nanoparticles in the CM and DEAE-sepharose depleted protein fractions than cell free extracts (Figure 5). CM-sepharose protein depleted fraction resulted bigger sized nanoparticles than DEAE-sepharose protein depleted fractions (Figure 5). These biosynthesized metallic nanoparticles showed size dependent collective oscillation of the conductance electron at different wavelengths and their crystalline nature (SADS, HRTEM, & Fourier image) is similar to that of the chemically synthesized SNPs [16,30]. A variety of silver nanoparticles (size and shape) are synthesized due to the amount and reactivity of proteinaceous and non-proteinaceous oxidoreductive species and surfactants present in the biological system [21,26,31] (Figure 2).

Biologically synthesized silver nanoparticles (SNPs) could have many applications in various fields but the uncontrolled synthesis (with respect to size and shape of SNPs) is major drawback for the commercial production of SNPs using the biological system. The evaluation of molecular mechanism of biosynthesis of SNPs is of prime importance to understand biosynthesis process and the development of methodology for controlling the shape and size (uniform distribution) of SNPs. In this report, the study is mainly focused to understand the role of diverse algal cellular proteins in the biosynthesis and capping of silver nanoparticles. Spectroscopic and SDS-PAGE analysis indicate the accumulation of various cellular proteins on *C. reinhardtii *mediated (*in vivo *and *in vitro) *SNPs biosynthesis. Proteins involved in the SNPs biosynthesis were identified by MALDI-MS-MS using mascot platform. Most of these SNPs bound proteins are from oxidoreductive machinery of *Chlamydomonas *cells. The major SNPs associated proteins are identified as ATPase, sedoheptulose-1,7-bisphosphatase, carbonic anhydrase, ferredoxin NADP^+ ^reductase (FNR), superoxide dismutase (SOD), oxygen evolving enhancer protein (OEE), ribulose bisphosphate carboxylase and nuclear histone (H4) (Table 1). Our experimental finding of histone association with biosynthesized SNPs indicates the role of *C. reinhardtii *histone (H4) in the SNPs synthesis and capping. Carbonic anhydrase (CA), Ferredoxin and Ferredoxin NADP^+ ^reductase (FNR) proteins are also found associated with synthesized SNPs. The histone (H4) is well known for the reduction of silver ammonia complex into ~20 nm SNPs and binds SNPs with lysine rich AKRHRK domain [32-34]. This histone mediated SNPs synthesis is due to redox reaction activity of this domain [35]. These proteins might act together as redox centre for converting silver ions into SNPs [36,37]. The involvement of NAD in SNPs synthesis has also been predicted by Ahamad et al. [38]. The binding of superoxide dismutase (SOD) with biosynthesized SNPs suggest the involvement of this protein in the reduction of Ag^+ ^to SNPs. Recently, it has been shown that superoxide (O_2_^--^) is not only involved in peroxide formation but is also able to reduce Ag^+ ^to Ag^0^. Further, this step might be sufficient for nucleation step in SNPs synthesis and may modulate rate of formation of SNPs [39]. Another SNPs associated protein *viz *sedoheptulose-1, 7-bisphosphatase (SBPase) might be involved in the capping of SNPs through its free -SH group, which is generated by thioredoxin mediated reduction [40,41]. Biosynthesized SNPs are mostly localized in cup-shaped chloroplast of *C. reinhardtii *(Figure 3), a major organ for photosynthetic machinery [42]. This machinery responds first to metal stress by over expressing proteins like ATP synthase, RuBP carboxylase and oxygen evolving enhancer protein (OEE) proteins [43-45]. These enzymes are found associated with *C. reinhardtii *mediated biosynthesized SNPs (Table 1).

Based on the current findings and preliminary evidence about the role of the cellular proteins for the biosynthesis of silver nanoparticles, it is accomplished that the SNPs biosynthesis is governed by various cellular proteins present in the system. The other small biomolecules may have potential to act as various oxidation-reduction systems for SNPs biosynthesis either separately or as supplementary to these proteins partners. It would be interesting to delineate the role of identified protein molecule(s) in the control of size and/or shape of the biosynthesized nanoparticles.

## Conclusions

Most of the eukaryotic organisms are reported to have potential to synthesize silver nanoparticles of different shape/size and synthetic rate depending on their proteinaceous and non- proteinaceous biochemical oxidoreductive species. Our work confirms the SNPs biosynthesis potential of *C. reinhardtii *as unicellular model system. Further study to evaluate the molecular mechanism of SNPs biosynthesis, we have found various cellular proteins *viz *histone (H4), CA, FNR, SOD, SBPase, ATP synthase, RuBP carboxylase, and OEE associated with biosynthesized SNPs. However, these proteins are not associated with the pre-synthesized silver nanoparticles. The alterations in size and biosynthesis rate of SNPs by proteins depleted fractions confirm that these proteins have direct control on *C. reinhardtii *mediated biosynthesis of SNPs. This is the first report of elucidation of molecular machinery and the role of oxidoreductive cellular proteins in the biosynthesis of silver nanoparticles.

## Methods

### Culture of the *C. reinhardtii*

*Chlamydomonas *strain CC-124 (green algae) was procured from *Chlamydomonas *centre, Duke University, U.S.A. The culture was maintained on agar plate by adding 1.5% agar in TAP media supplemented with Hutner's trace element solution. Culture was grown in TAP media under optimum light conditions i.e, 2300 lux. Cells were collected by centrifugation at 3500 rpm for 5.0 min at room temperature and washed properly with distilled water (DW).

### Preparation of *C. reinhardtii *cell free extract for nanoparticle biosynthesis

Typically, 2 g (wet weight) biomass of *C. reinhardtii *was suspended in 20 ml of double distilled sterile water for 2 h at 27°C in an Erlenmeyer flask with gentle agitation followed by vortexing with glass beads. To avoid heating due to the vigorous mixing, the cells were agitated for 1 min, followed by a 30 s cooling period between agitation cycles. Finally, the cells were broken by intermittent sonication (2 second on and 10 second off pulse) at 4°C in ice for 10 min at 40% amplitude. The cell suspension was examined microscopically until most of the cells were broken. Cell free filtrate was obtained by centrifugation of the *C. reinhardtii *at 10000 rpm for 10 min at 4°C. This solution (10% wet w/v) was used for the further experiments.

### Silver nanoparticles synthesis

#### In vitro

The SNPs synthesis reaction was carried out by incubating 10% (*v*/*v*) of cell free extract with 1.0 mmol L^-1 ^aqueous AgNO_3 _(double distilled water) solution for 15 days at room temperature in dark. The change in color of the whole solution was an indication of the synthesis of SNPs. The solution was centrifuged at 12000 rpm for 20 min to separate the SNPs. These SNPs were washed five times with double distilled water and separated from reaction mixture along with the associated proteins.

#### In vivo

The optimally grown culture (100 ml) was centrifuged (3000 rpm) for 5 min in sterile condition, washed three times to remove the culture medium and resuspended in 50 ml autoclaved water. The stock solution of AgNO_3 _(autoclaved) in water was added to make the final 1 mM AgNO_3 _solution. One ml reaction solution was harvested, sonicated, filtered to remove cell debris, centrifuged, washed and resuspended in 200 μl of distilled water at different time intervals (Figure 1) to study the kinetics of SNPs synthesis. The absorbance scan was taken for the entire sample and the biosynthesized SNPs were characterized electron microscopically/elemental/crystallinity for their morphology using TEM, EDAX and HRTEM image respectively.

### Spectroscopic and kinetic characterization of silver nanoparticles

The reduction of silver ions was monitored by measuring absorbance scan at 250-700 nm at selected time intervals with UV-VIS spectrophotometer (Nano Drop) (Figure 1). Change in color was observed in the silver nitrate solution incubated with the *C. reinhardtii *cell free extract. The silver nanoparticles dispersed in water were kept at room temperature (37°C) and the absorption at 470 nm was measured continuously to determine their stability.

The conversion of AgNO_3 _into SNPs were determined indirectly on Inductively Coupled Plasma-Mass Spectrometry (ICP-MS) by measuring the remaining Ag+ concentration in supernatant after removal of biosynthesized SNPs (by centrifugation) at various time interval (0, 0.5, 1, ...10 h). Briefly, 4 ml of the SNPs free supernatant was dried at 105°C for 3 h. After addition of 4 ml of concentrated nitric acid and 0.5 ml of concentrated hydrochloric acid, the samples were digested by using a microwave power progressively increasing up to 400 W for 40 min. After cooling, the solutions were accurately diluted to 50 ml with water. One replicate per digestion method was done for each sample and they were analyzed directly by ICP-MS.

### SDS-PAGE gel analysis of SNPs associated proteins

Thoroughly washed C. *reinhardtii *cellular proteins associated with SNPs were heated at 95°C for 5 min to dissociate the cellular proteins from SNPs. The SNPs were separated from the solution by centrifugation at 14000 rpm. The protein content of this solution was quantified by Bradford assay. 100 μg (as determined using the Bradford assay) of the soluble proteins were heated at 95°C for 5 min in 4% β-mercaptoethanol and 2% SDS. Proteins associated with the SNPs were resolved on 12% SDS-PAGE.

### Sample preparation for proteomics

Protocol for sample preparation for proteomics analysis of SNPs associated proteins was adopted from published papers [46,47]. Briefly, protein bands on SDS-PAGE were excised, de-stained, washed with water and dehydrated with acetonitrile (ACN). Proteins were reduced with 10 mM DTT (dithiothreitol) solution in trypsin digestion buffer at 60°C for 30 min. After cooling at room temperature, gels were dehydrated with ACN. The proteins were alkylated with 50 mM iodoacetamide solution in trypsin digestion buffer in dark as per manufacturer's instruction. After washing with ACN, gel samples were dried in speedvac. Gel pieces were rehydrated with trypsin rehydration solution containing 50 ng of trypsin and incubated at 37°C overnight for in gel digestion of protein. Peptides were recovered with Pep-extract buffer (supplied with kit), lyophilized, and reconstituted in 10 μl of 50% ACN and 0.1% trifluoroacetic acid (TFA).

### MALDI MS-MS analysis of SNPs bound proteins

MALDI experiments were performed on pre-calibrated ABI 4800 *plus *MALDI-TOF-TOF analyzer (Applied Biosystem) at department of Biochemistry, Delhi University South Campus, New Delhi, India. Reconstituted peptides were spotted on 384 well LC MALDI stainless steel plate. For MS analysis, spots were illuminated with laser intensity of 3500 and total 1200 spectra were recorded [48,49]. For MS-MS analysis, 25 precursors/peptides were selected from each spot and depending upon their abundance, each precursor was fragmented in the collision cell. Peptide fragmented with 4300 laser intensity and 2500 shots were accumulated for processing of data [50,51].

The resultant PMF and peptide fragments were searched against swissprot database on MASCOT search engine http://www.matrixscience.com. The search of database was focused for *Virdiplantae *group containing green algae and land plant genome database. The monoisotopic peptide tolerance was kept at ± 100 ppm with MS/MS tolerance of ± 0.6 Da.

### Protein depletion experiment

The protein depletion experiment was carried out to confirm the role of various proteins in the biosynthesis of silver nanoparticles. The *C. reinhardtii *cell free extract (5 ml) was passed through pre-equilibrated CM-sepharose and DEAE-sepharose ion exchange chromatographic column at pH 7.0. The flow through of each column was used for the biosynthesis of silver nanoparticles and to characterize their synthesis kinetics. Morphological comparisons of the biosynthesized SNPs were characterized by using TEM.

### Protein binding assay

Protein binding assay was performed to confirm the involvement of various proteins either in the synthesis or capping or both. For this purpose, freshly synthesized SNPs (without any protein capping) were incubated with *C. reinhardtii *cell free extract for three days in standard conditions. These nanoparticles were separated, gently washed and heated with 0.2% SDS to dissolve all the bound protein on nanoparticles. The supernatant after the SDS treatment was resolved on the SDS PAGE.

### Electron microscopic characterization of SNPs

#### SEM imaging

Biosynthesis of silver nanoparticles *in vivo *was characterized by scanning electron microscope (S-3400 N, Hitachi, Japan). The AgNO_3 _incubated *C. reinhardtii *cultures at different time (0, 1, 5, and 8 h) were mounted on an aluminum stub using double sided carbon tape. The completely dried sample was coated with gold by sputter coating unit (E1010 ion sputter Hitachi, Japan) and image was captured on SEM mode at desired magnification.

#### TEM, diffraction and EDAX characterization of nanoparticles

The morphology and grain size of the *C. reinhardtii *biosynthesized silver nanoparticles were observed by high-resolution transmission electron microscopy using FEI G-20 model operated at 200 kV. The crystallinity of the nanoparticles was determined using selected area electron diffraction (SAED) coupled with TEM. Elemental analysis of the nanoparticles was carried out using a scanning transmission electron microscope (STEM) equipped with an energy dispersive X-ray (EDX) spectrometer (EDAX). Briefly, the washed biosynthesized SNPs were directly spread on grid and dried before TEM imaging. However, the cells were fixed with glutaraldehyde, OsO4 and embedded in resins after serial alcohol dehydration for exploring the cellular localization of *in vivo *synthesized SNPs. The ultrathin sections (~60 nm) of the cells were used for the TEM imaging.

## Competing interests

The authors declare that they have no competing interests.

## Authors' contributions

IB did the culturing of *C. reinhardtii*, biosynthesis kinetics, protein depletion and SDS PAGE analysis and PR did the MALDI-TOF-TOF and protein identification. SK and SC wrote the manuscript and design the various experiments. SC performed the electron microscopic analysis using TEM and SEM. All the authors has read and approved this final manuscript for publication.

## Supplementary Material

Additional file 1 **UV visible scan (a) *in vitro *and (b) *in vivo *system SNPs biosynthesis**. This file shows the absorbance scan at various time (given along with figure) in vitro and in vivo conditions. Click here for file

Additional file 2 **Cellular localization of *in vivo *synthesized silver nanoparticles**. (a) TEM micrograph SNPs synthesizing cell at different time and (b) SEM image of 1 mM AgNO_3 _incubated *C. reinhardtii cell *at different time period (given in the right corner of each image). Click here for file

Additional file 3 **Cellular localization of silver nanoparticles inside the cell**. Various TEM micrograph of thin section (~60 nm) of in vivo biosynthesized SNPs containing cells for better conspicuousness. Click here for file

Additional file 4 **SDS-PAGE of equal volume (50 and 100 μl) of *C. reinhardtii *depleted proteins**. Cell free extract (lane 1-2), DEAE-Spharose depleted flow through (lane 3-4), DEAE 0.5 M salt wash (lane 5-6), CM-sepharose depleted flow through (lane 7-8), and CM-sepharose 0.5 M salt wash (lane 9-10). Click here for file

Additional file 5 **Absorbance scans of protein depleted fractions**. The absorbance scans of *C. reinhardtii cell *free extract, DEAE-Spharose, CM-sepharose depleted flow through samples before AgNO_3 _incubation. Click here for file

Additional file 6 **PMF (upper panel) and MS-MS (lower panel) spectra of SNPs associated various identified proteins**. (a) ATP synthase subunit alpha, chloroplastic OS = *Chlamydomonas reinhardtii (b) *ATP synthase subunit beta, chloroplastic (c) Carbonic anhydrase (d) Sedoheptulose-1,7-bisphosphatase (e) Ferredoxin, chloroplastic (f) Oxygen-evolving enhancer protein 1, chloroplastic (g) Oxygen-evolving enhancer protein 2, chloroplastic (h) Oxygen-evolving enhancer protein 3 (i) Histone H4. *de novo *sequence of the peptides was given along with each MS-MS spectra in red. Click here for file
