# Supplementary figures and images for: Cellular oxido-reductive proteins of Chlamydomonas reinhardtii control the biosynthesis of silver nanoparticles
Source: J Nanobiotechnology. 2011 Dec 7;9:56. doi: 10.1186/1477-3155-9-56 (PMC3283517; doi:10.1186/1477-3155-9-56)

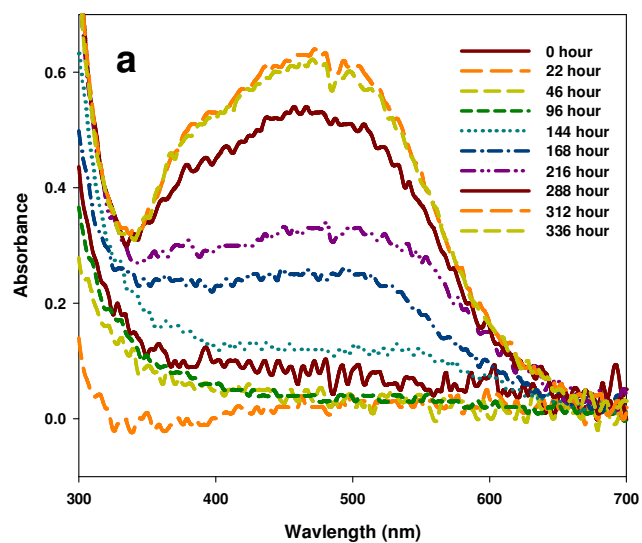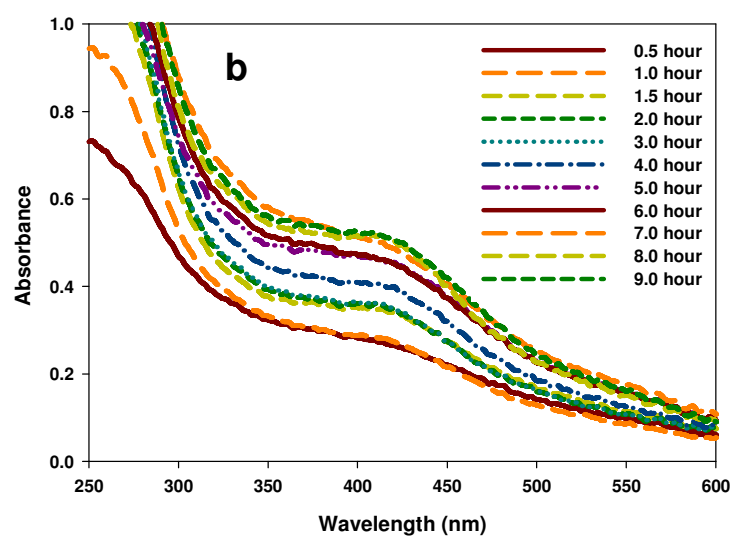

Supplement: Additional file 1 — UV visible scan (a) in vitro and (b) in vivo system SNPs biosynthesis. This file shows the absorbance scan at various time (given along with figure) in vitro and in vivo conditions. [file 1477-3155-9-56-S1.PDF]

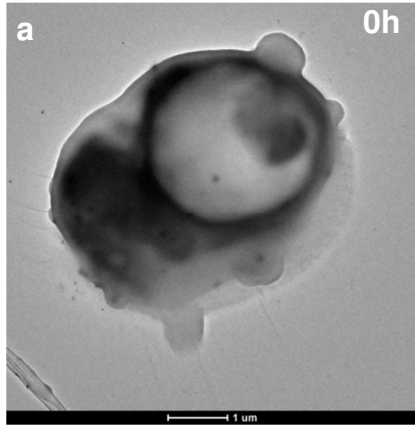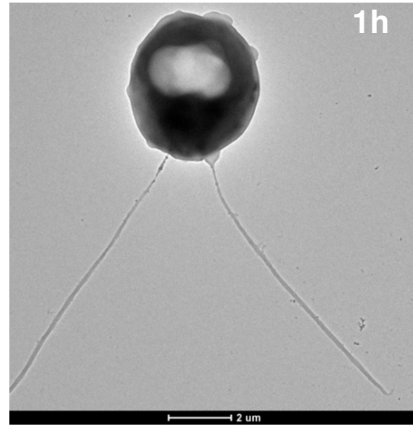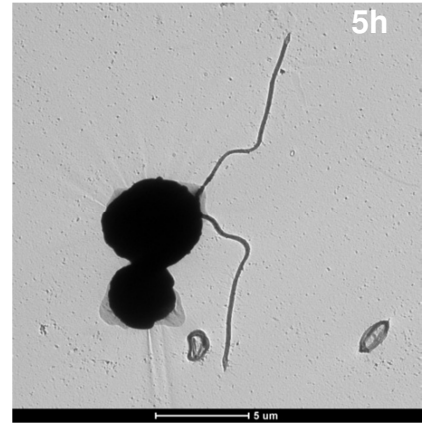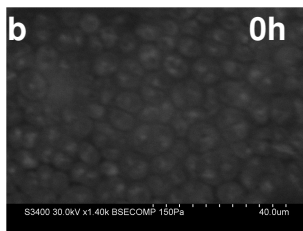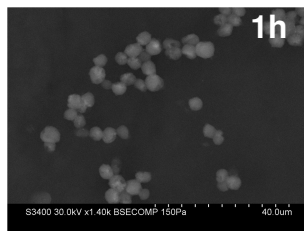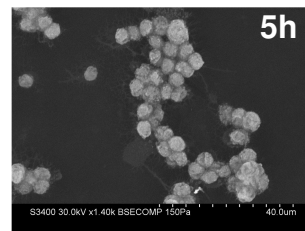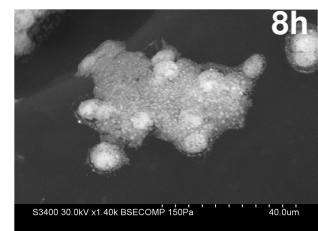

Supplement: Additional file 2 — Cellular localization of in vivo synthesized silver nanoparticles. (a) TEM micrograph SNPs synthesizing cell at different time and (b) SEM image of 1 mM AgNO3 incubated C. reinhardtii cell at different time period (given in the right corner of each image). [file 1477-3155-9-56-S2.PDF]

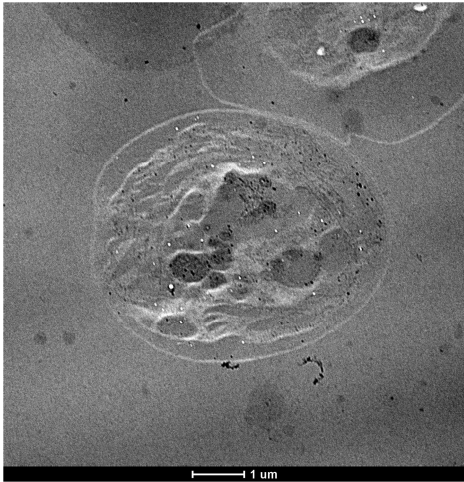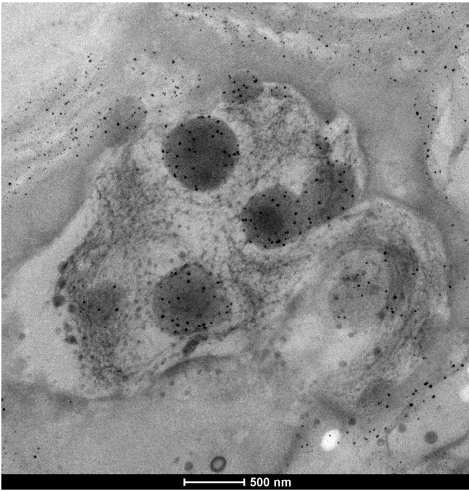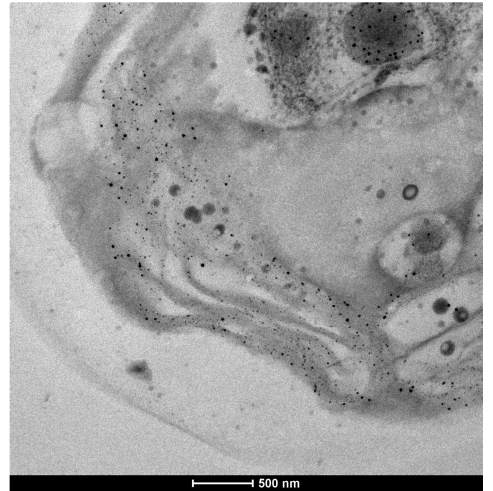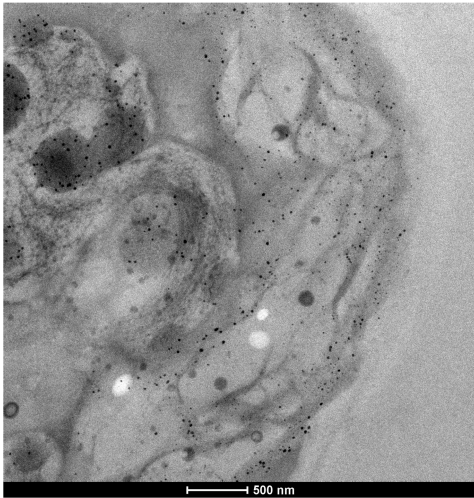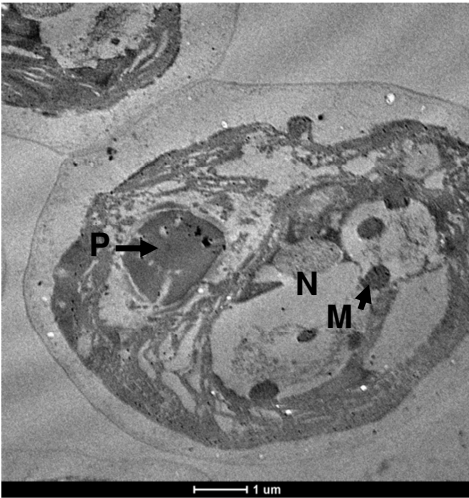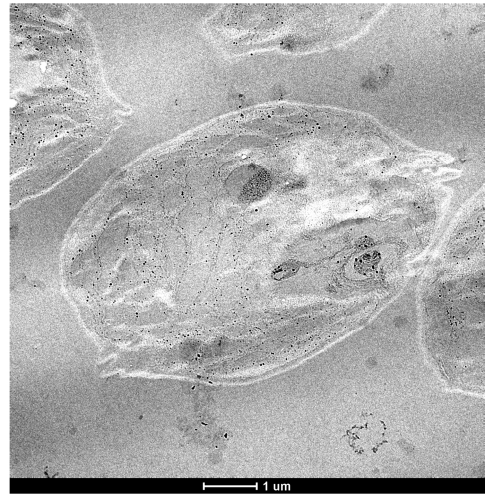

Supplement: Additional file 3 — Cellular localization of silver nanoparticles inside the cell. Various TEM micrograph of thin section (~60 nm) of in vivo biosynthesized SNPs containing cells for better conspicuousness. [file 1477-3155-9-56-S3.PDF]

1 2 3 4 5 6 7 8 9 10

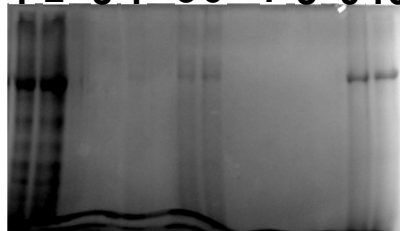

Supplement: Additional file 4 — SDS-PAGE of equal volume (50 and 100 μl) of C. reinhardtii depleted proteins. Cell free extract (lane 1-2), DEAE-Spharose depleted flow through (lane 3-4), DEAE 0.5 M salt wash (lane 5-6), CM-sepharose depleted flow through (lane 7-8), and CM-sepharose 0.5 M salt wash (lane 9-10). [file 1477-3155-9-56-S4.PDF]

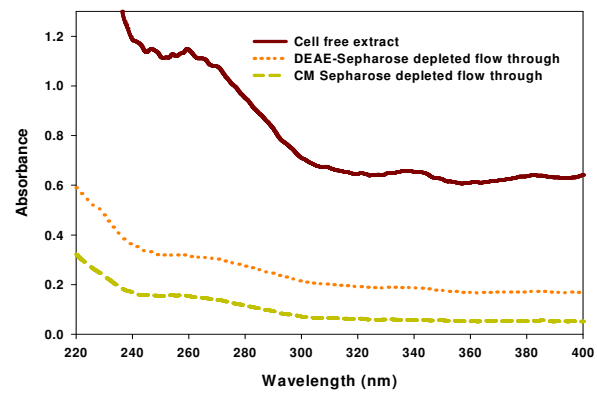

Supplement: Additional file 5 — Absorbance scans of protein depleted fractions. The absorbance scans of C. reinhardtii cell free extract, DEAE-Spharose, CM-sepharose depleted flow through samples before AgNO3 incubation. [file 1477-3155-9-56-S5.PDF]
