# Supplementary material for: Cellular oxido-reductive proteins of Chlamydomonas reinhardtii control the biosynthesis of silver nanoparticles
Source: J Nanobiotechnology. 2011 Dec 7;9:56. doi: 10.1186/1477-3155-9-56 (PMC3283517; doi:10.1186/1477-3155-9-56)
Supplement: Additional file 6 — PMF (upper panel) and MS-MS (lower panel) spectra of SNPs associated various identified proteins. (a) ATP synthase subunit alpha, chloroplastic OS = Chlamydomonas reinhardtii (b) ATP synthase subunit beta, chloroplastic (c) Carbonic anhydrase (d) Sedoheptulose-1,7-bisphosphatase (e) Ferredoxin, chloroplastic (f) Oxygen-evolving enhancer protein 1, chloroplastic (g) Oxygen-evolving enhancer protein 2, chloroplastic (h) Oxygen-evolving enhancer protein 3 (i) Histone H4. de novo sequence of the peptides was given along with each MS-MS spectra in red. [file 1477-3155-9-56-S6.PDF]

(a)

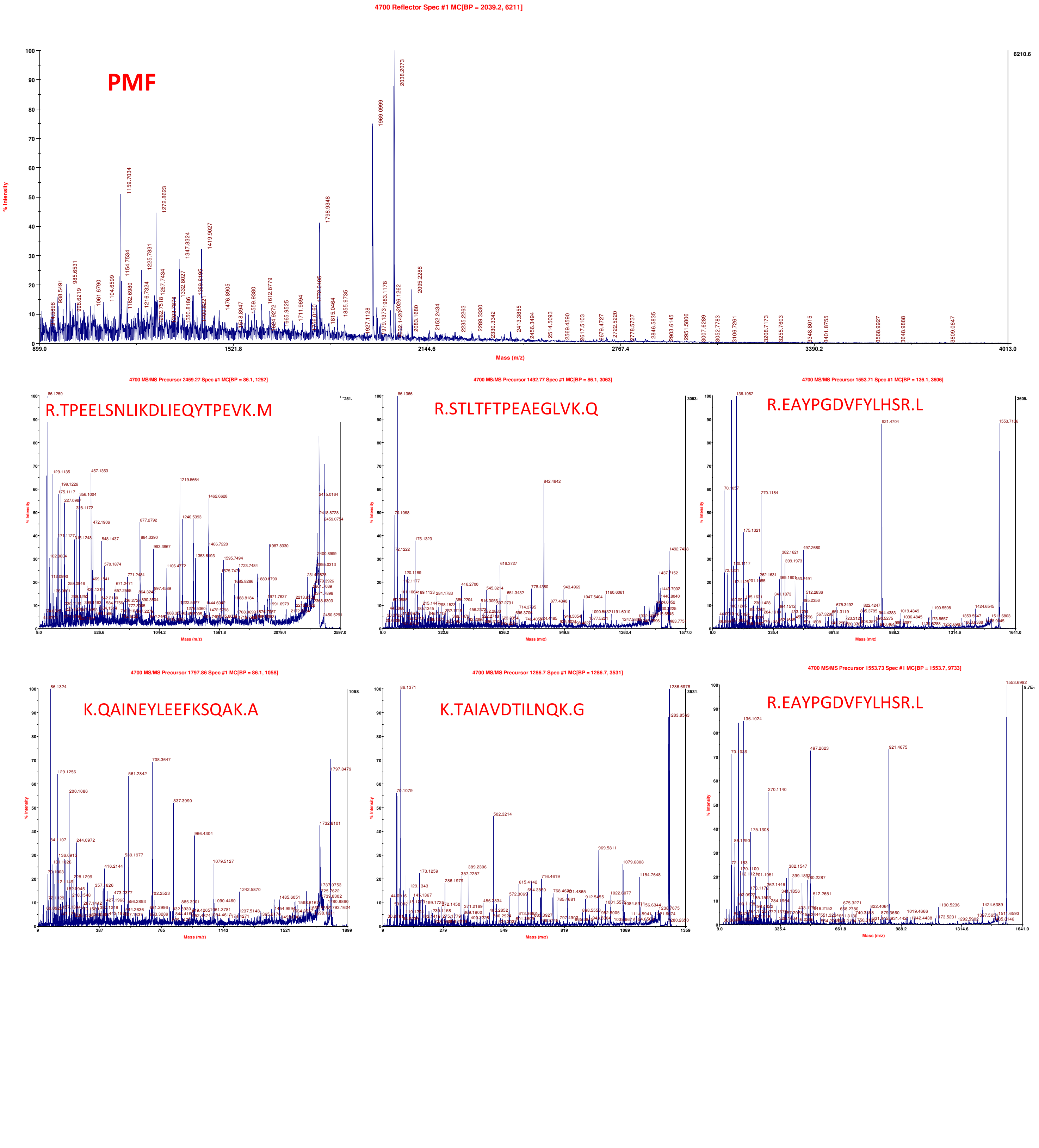
4700 Reflector Spec #1 MC[BP = 2039.2, 6211]
PMF
% Intensity
Mass (m/z)
4700 MS/MS Precursor 2459.27 Spec #1 MC[BP = 86.1, 1252]
R.TPEELSNLIKDLIEQYTPEVK.M
4700 MS/MS Precursor 1492.77 Spec #1 MC[BP = 86.1, 3063]
R.STLTFTPEAEGLVK.Q
4700 MS/MS Precursor 1553.71 Spec #1 MC[BP = 136.1, 3606]
R.EAYPGDVFYLHSR.L
4700 MS/MS Precursor 1797.86 Spec #1 MC[BP = 86.1, 1058]
K.QAINEYLEEFKSQAK.A
4700 MS/MS Precursor 1286.7 Spec #1 MC[BP = 1286.7, 3531]
K.TAIAVDTILNQK.G
4700 MS/MS Precursor 1553.73 Spec #1 MC[BP = 1553.7, 9733]
R.EAYPGDVFYLHSR.L

(b)

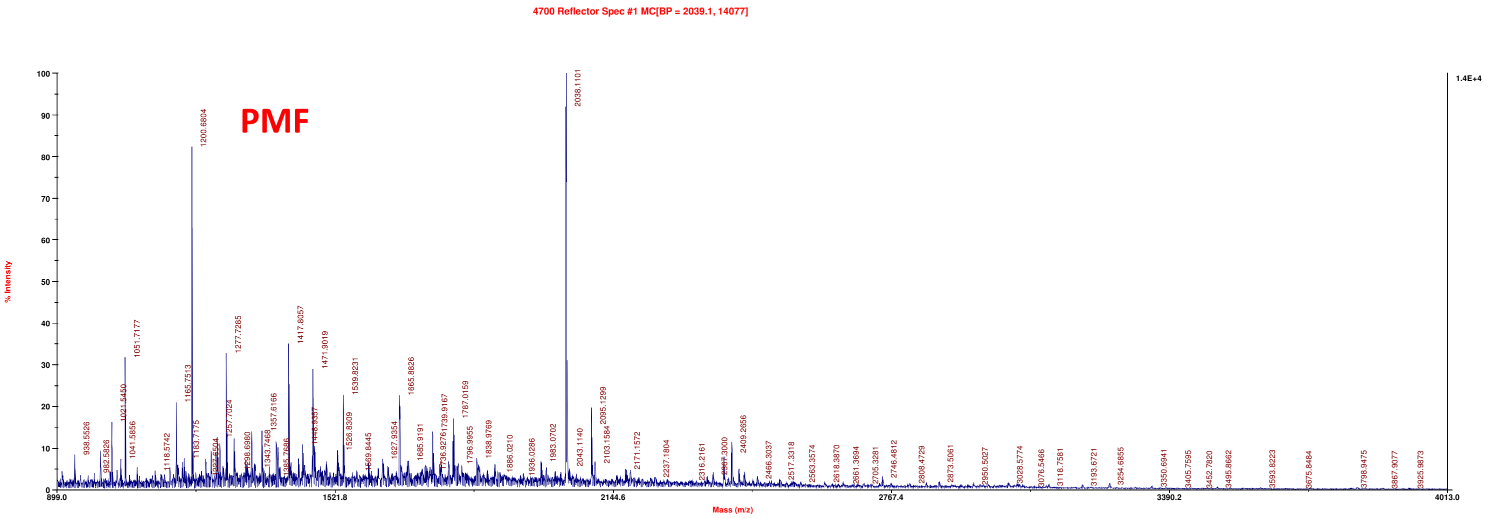
4700 Reflector Spec #1 MC[BP = 2039.1, 14077]
PMF
% Intensity
Mass (m/z)
1.4E+4
2038.1101
1200.6804
1051.7177
1277.7285
1417.8057
1471.9019
1539.8231
1665.8826
1021.5450
1041.5856
1165.7513
1183.7175
1257.7024
1357.6166
1343.7485
1238.6980
1526.8309
1627.9354
1685.9191
1739.9167
1787.0159
1796.9955
1838.9769
1886.0210
1936.0286
1983.0702
2043.1140
2095.1299
2103.1584
2171.1572
2237.1804
2316.2161
2409.2656
2466.3037
2517.3318
2563.3574
2618.3870
2661.3694
2705.3281
2746.4812
2808.4729
2873.5061
2950.5027
3028.5774
3076.5466
3118.7581
3193.6721
3254.6855
3350.6941
3405.7595
3452.7820
3495.8662
3593.8223
3675.8484
3798.9475
3867.9077
3925.9873
938.5526
982.5826
1118.5742
899.0
1521.8
2144.6
2767.4
3390.2
4013.0

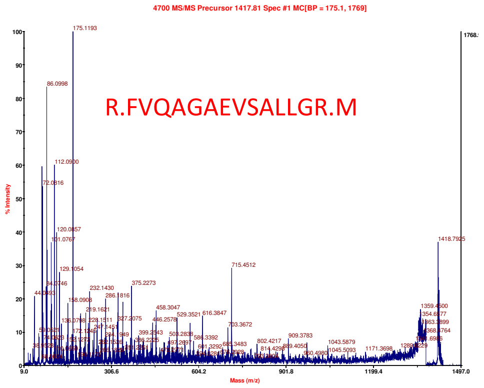
4700 MS/MS Precursor 1417.81 Spec #1 MC[BP = 175.1, 1769]
R.FVQAGAEVSALLGR.M
% Intensity
Mass (m/z)
175.1193
86.0998
112.0900
72.0816
120.0857
101.0767
129.1054
1418.7925
715.4512
375.2273
232.1430
1359.4500
9.0
306.6
604.2
901.8
1199.4
1497.0

(c)

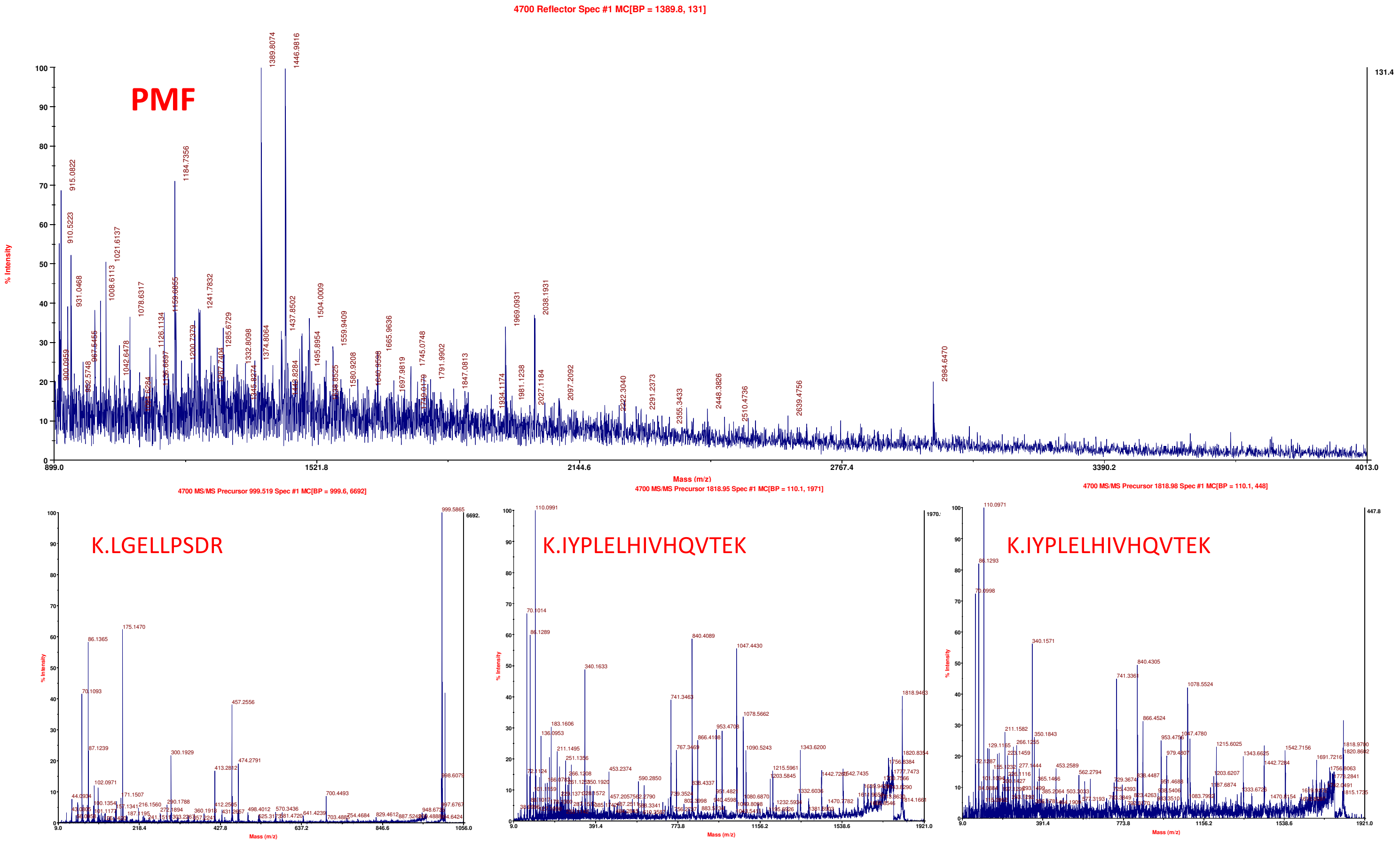
4700 Reflector Spec #1 MC[BP = 1389.8, 131]
PMF
% Intensity
Mass (m/z)
1389.8074
1446.9816
1184.7356
915.0822
910.5223
1021.6137
2038.1931
1969.0931
2984.6470
4700 MS/MS Precursor 999.519 Spec #1 MC[BP = 999.6, 6692]
K.LGELLPSDR
999.5865
175.1470
86.1365
457.2556
4700 MS/MS Precursor 1818.95 Spec #1 MC[BP = 110.1, 1971]
K.IYPLELHIVHQVTEK
110.0991
840.4089
1047.4430
340.1633
1818.9463
4700 MS/MS Precursor 1818.98 Spec #1 MC[BP = 110.1, 448]
K.IYPLELHIVHQVTEK
110.0971
340.1571
840.4305
741.3363
1078.5524

(d)

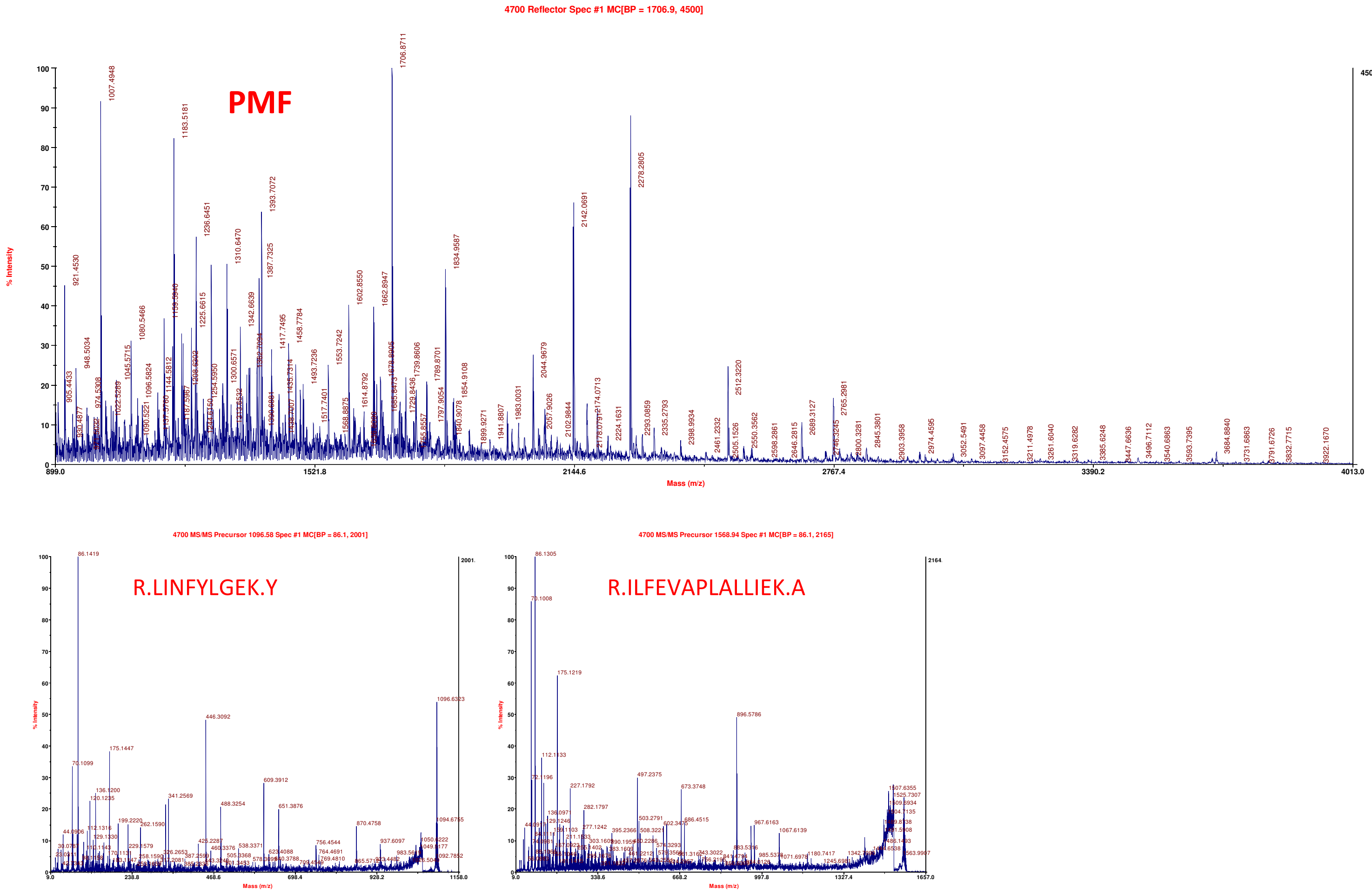
4700 Reflector Spec #1 MC[BP = 1706.9, 4500]
PMF
% Intensity
Mass (m/z)
4700 MS/MS Precursor 1096.58 Spec #1 MC[BP = 86.1, 2001]
R.LINFYLGEK.Y
4700 MS/MS Precursor 1568.94 Spec #1 MC[BP = 86.1, 2165]
R.ILFEVAPLALLIEK.A

(e)

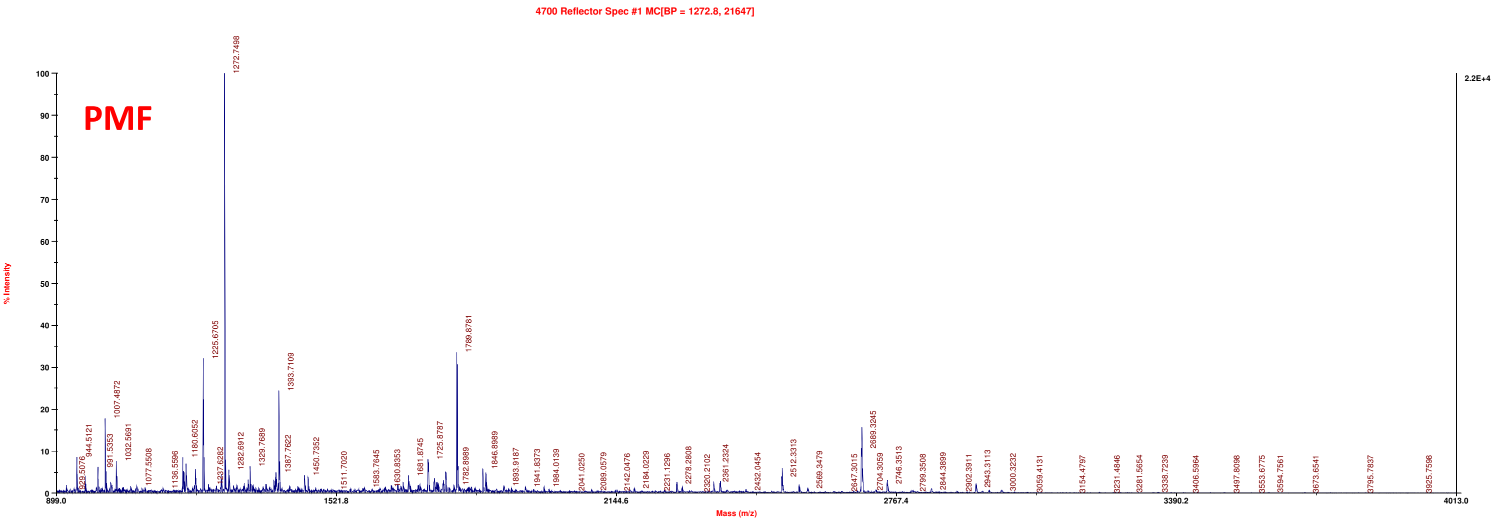
4700 Reflector Spec #1 MC[BP = 1272.8, 21647]
PMF
% Intensity
Mass (m/z)
2.2E+4
1272.7498
1225.6705
1789.8781
1393.7109
1007.4872
2689.3245

(f)

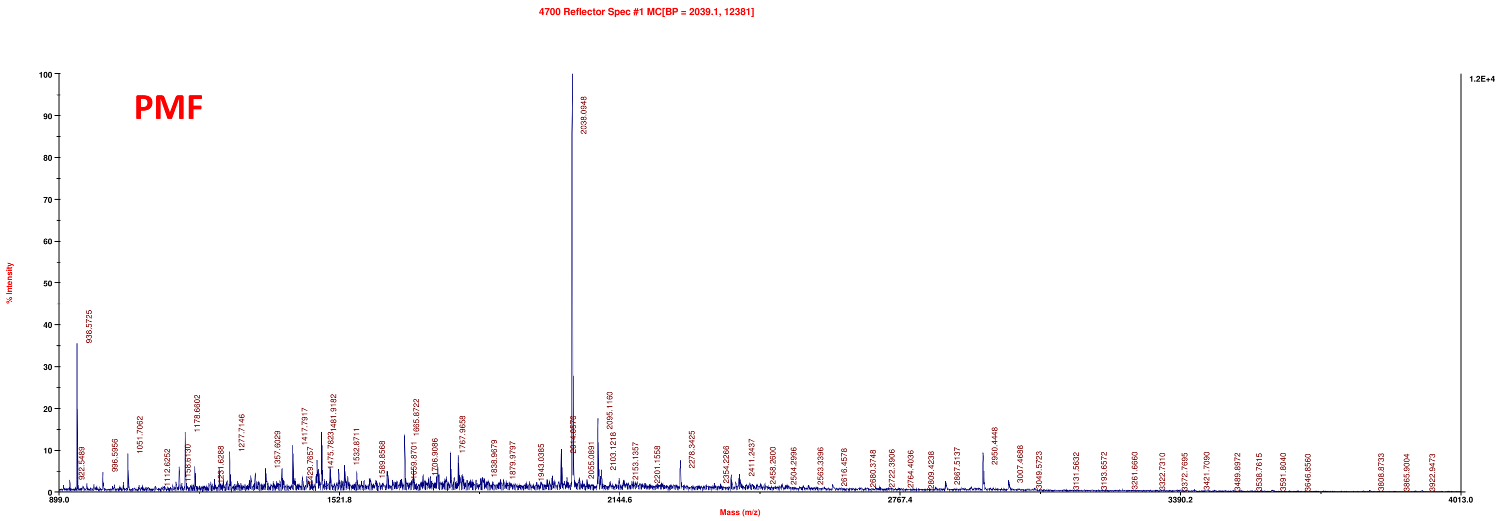
4700 Reflector Spec #1 MC[BP = 2039.1, 12381]
PMF
% Intensity
Mass (m/z)
1.2E+4
2038.0948
938.5725
1178.6602
2095.1160

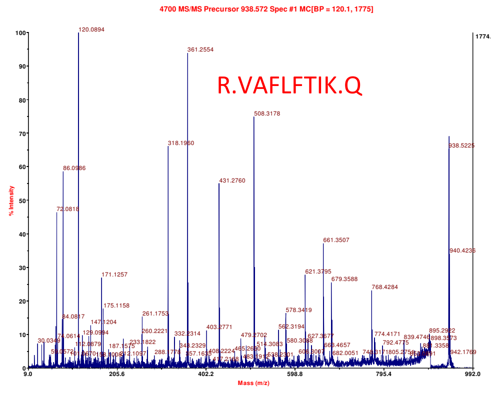
4700 MS/MS Precursor 938.572 Spec #1 MC[BP = 120.1, 1775]
R.VAFLFTIK.Q
% Intensity
Mass (m/z)
120.0894
361.2554
508.3178
938.5225

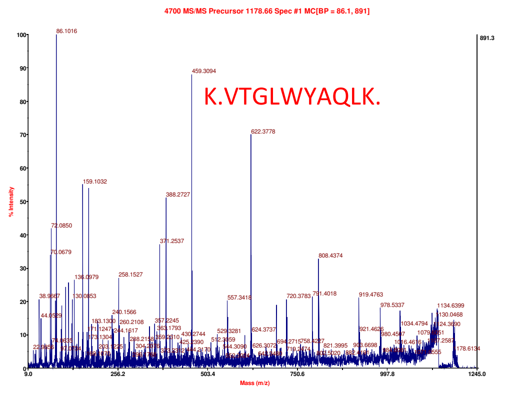
4700 MS/MS Precursor 1178.66 Spec #1 MC[BP = 86.1, 891]
K.VTGLWYAQLK.
% Intensity
Mass (m/z)
86.1016
459.3094
622.3778

(g)

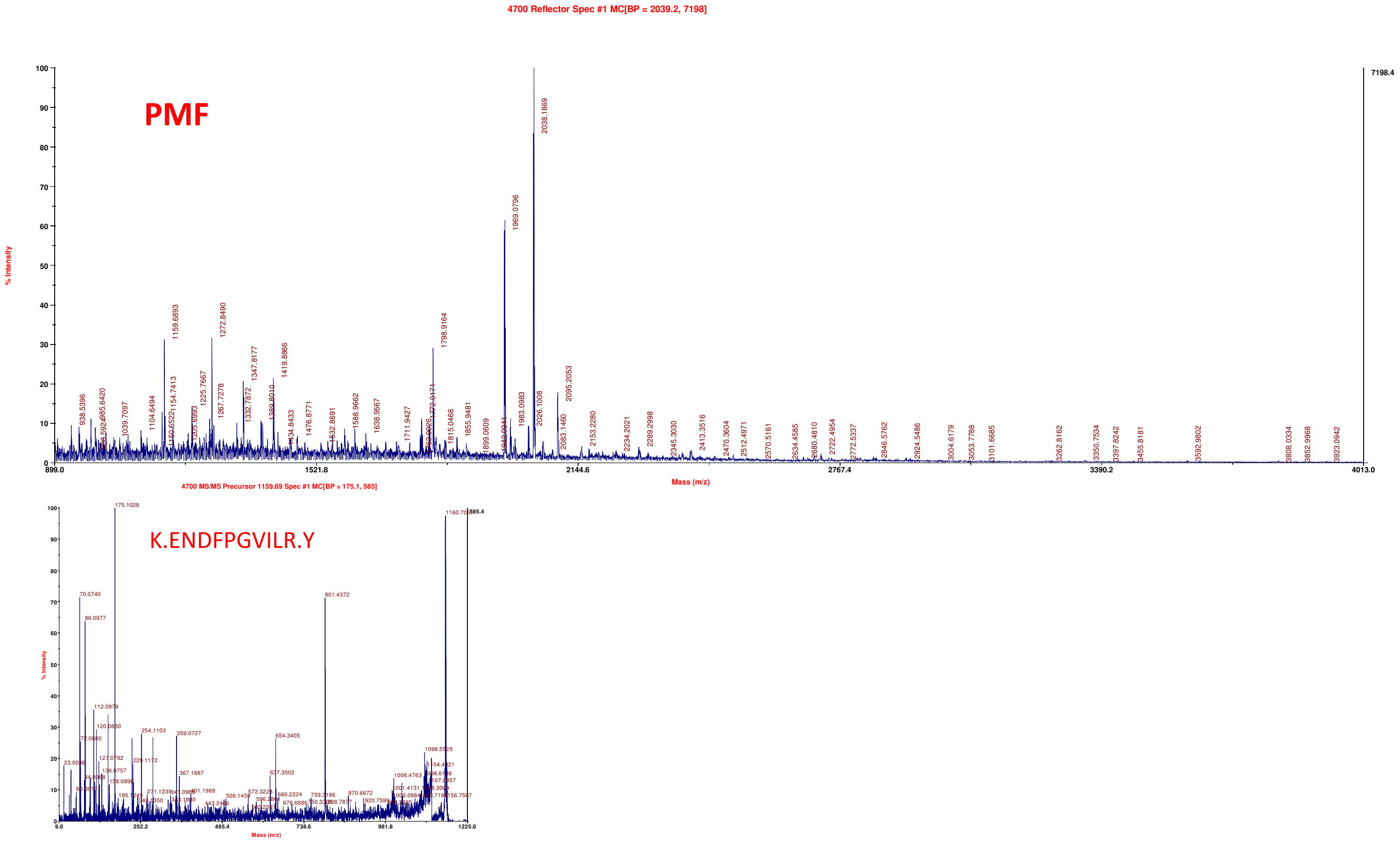
4700 Reflector Spec #1 MC[BP = 2039.2, 7198]
PMF
% Intensity
Mass (m/z)
7198.4
2038.1869
1969.0796
1798.9164
1272.8490
1159.6893
4700 MS/MS Precursor 1159.69 Spec #1 MC[BP = 175.1, 585]
K.ENDFPGVILR.Y
175.1028
801.4372
70.0740
86.0977
654.3405
585.4

(h)

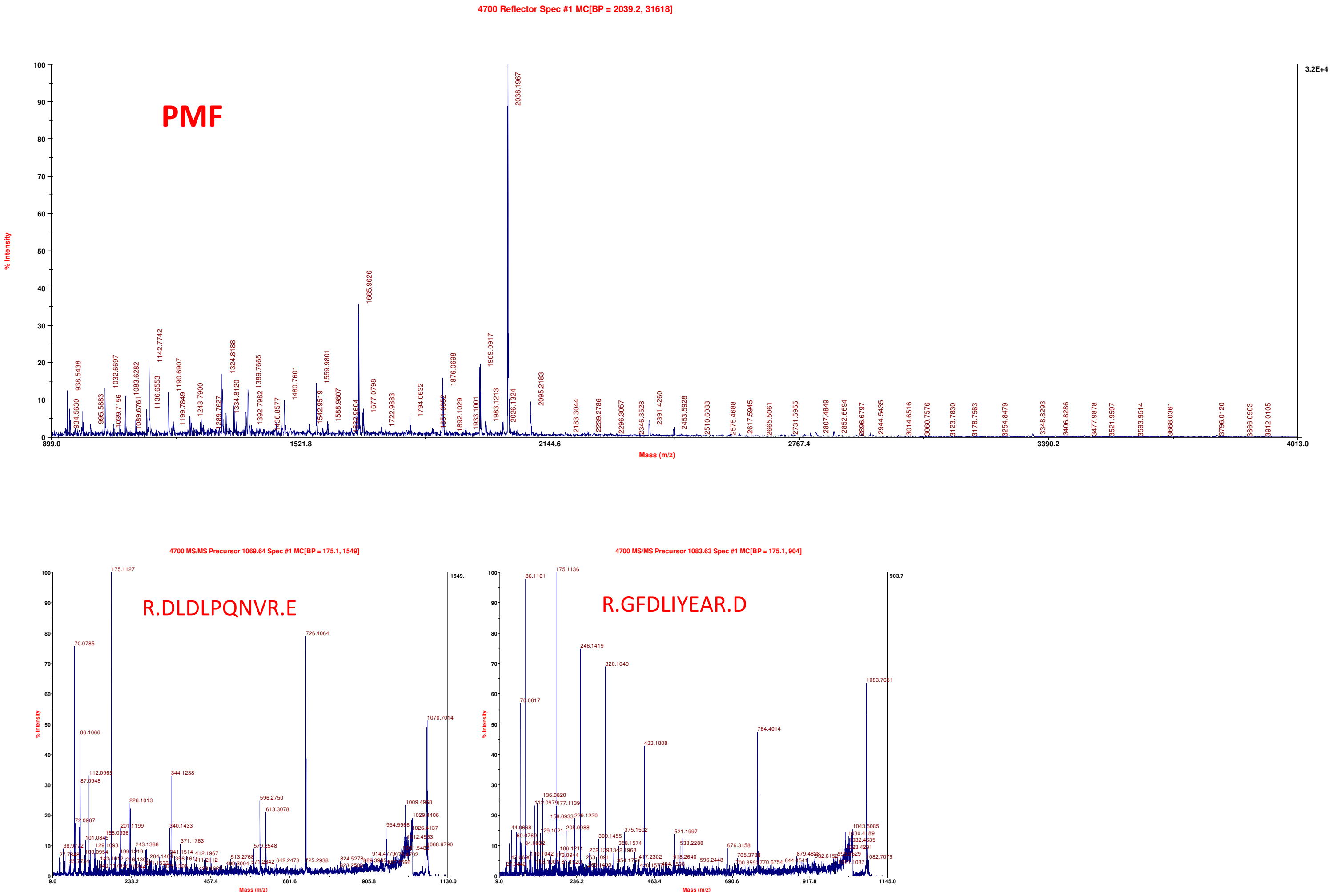
4700 Reflector Spec #1 MC[BP = 2039.2, 31618]
PMF
% Intensity
Mass (m/z)
3.2E+4
2038.1967
1665.9626
4700 MS/MS Precursor 1069.64 Spec #1 MC[BP = 175.1, 1549]
R.DLDLPQNVR.E
175.1127
726.4064
70.0785
1070.7014
4700 MS/MS Precursor 1083.63 Spec #1 MC[BP = 175.1, 904]
R.GFDLIYEAR.D
175.1136
86.1101
246.1419
320.1049
764.4014
433.1808

(i)

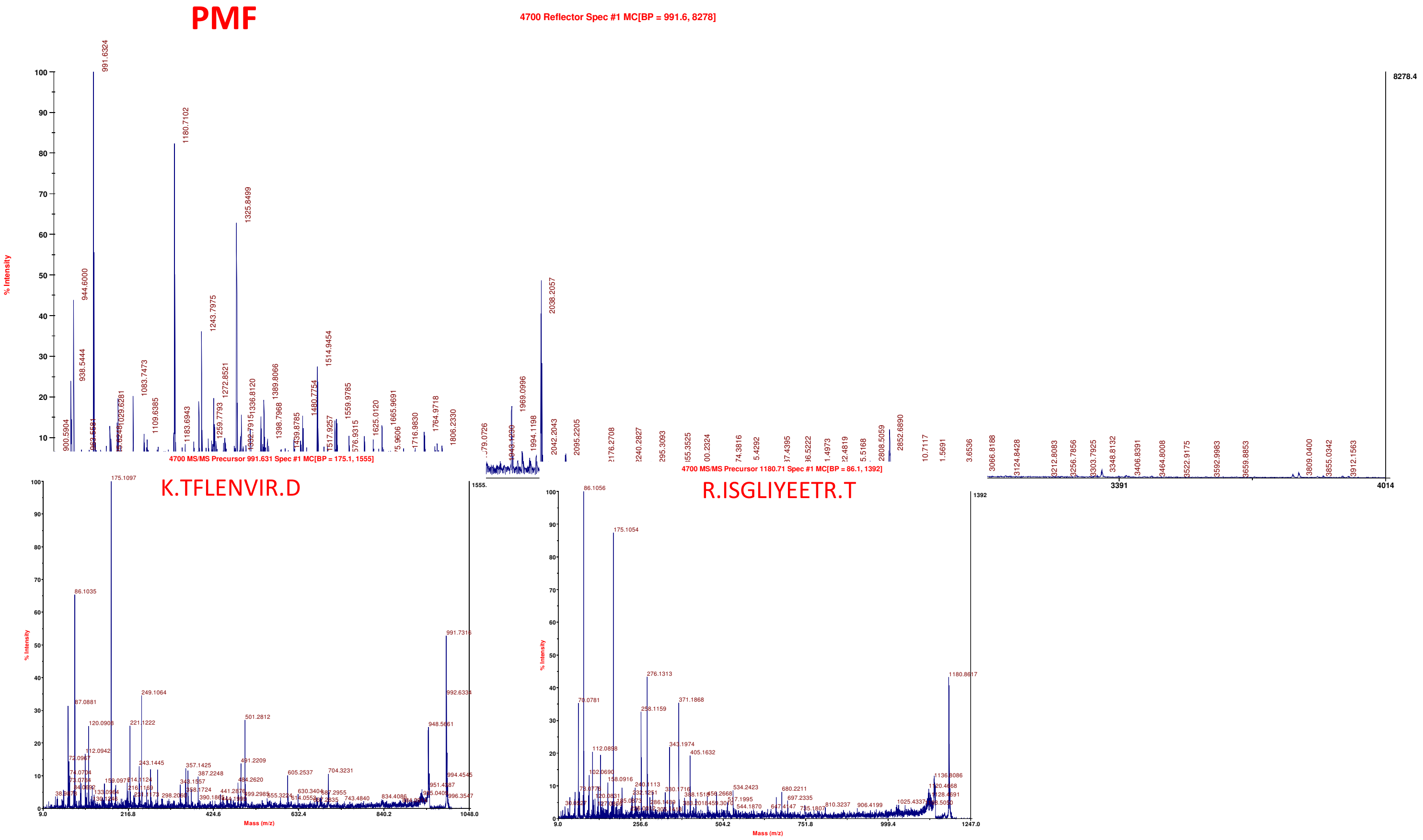
PMF
4700 Reflector Spec #1 MC[BP = 991.6, 8278]
% Intensity
8278.4
4700 MS/MS Precursor 991.631 Spec #1 MC[BP = 175.1, 1555]
K.TFLENVIR.D
4700 MS/MS Precursor 1180.71 Spec #1 MC[BP = 86.1, 1392]
R.ISGLIYEETR.T
Mass (m/z)
